# Radiological Dose Assessment to Members of the Public Using Consumer Products Containing Naturally Occurring Radioactive Materials in Korea

**DOI:** 10.3390/ijerph18147337

**Published:** 2021-07-09

**Authors:** Soja Reuben Joseph, Juyoul Kim

**Affiliations:** Department of Nuclear Power Plant Engineering, KEPCO International Nuclear Graduate School, 658-91 Haemaji-ro Seosaeng-myeon, Ulju-gun, Ulsan 45014, Korea; sojareuben@gmail.com

**Keywords:** radioactive consumer products, dose assessment, internal and external exposure, ICRP, NORM, VMC, IMBA, Microshield

## Abstract

Various products containing a small number of added radionuclides are commonly available for use worldwide. However, frequent use of such products puts the public at risk of radiation exposure. In this study, dose assessments to members of the public using consumer products containing naturally occurring radioactive materials (NORMs) were conducted for various usage scenarios to evaluate the external and internal exposure dose. Data for this study were obtained from previous literature and were statistically analyzed using Boxplot to determine the input data for assessment. A normalized value of activity concentration was used for dose evaluation. In addition to other external and internal dose calculation codes, analytical calculations were used to perform age-dependent. Based on analytical calculations, the highest total effective dose equivalent (TEDE) received from necklace products at the upper whiskers with an activity concentration of 4.21 Bq/g for ^238^U, 24.4 Bq/g for ^232^Th, and 0.55 Bq/g for ^40^K for various age groups is 2.03 mSv/y for 1 year old, 1.24 mSv/y for 10 years old and 1.11 mSv/y for adult, which are above the international commission for radiation protection (ICRP) recommended public dose limit of 1 mSv/y. Results of external and internal exposure dose obtained using Microshield code, IMBA code and Visual Monte Carlo (VMC) code are all below the recommended public dose limit of 1 mSv/y.

## 1. Introduction

Long-lived radionuclides are abundant in all rocks and minerals on earth, and many of them belong to the thorium (^232^Th) and uranium (^238^U) decay series. The majority of naturally occurring radionuclides belong to the radionuclides in the ^238^U and ^232^Th series, and the single decay radionuclide, ^40^K. Naturally occurring radioactive material (NORMs) are materials that contain such radionuclides [1]. Uranium and thorium are known as primordial radionuclides because they are available in all raw materials in varying concentrations, and are sources of natural background radiation in our environment. Combination of uranium and thorium in any physical or chemical form, or ore containing 0.05% or more by weight of uranium and thorium can be termed as source materials [2]. The activity concentration of these radionuclides in raw materials is low, but it can increase significantly above the usual background level after processing, which raises radiological risk and potentially exposing the public to radiation. The International Atomic Energy Agency (IAEA) recommends that member states provide NORMs industry workers with safety measures to reduce the risk of excessive radiation exposure [3]. Workers in NORMs industries are exposed to large amounts of radiation emitted by bulk quantities of NORMs during manufacturing processes, which can lead to significant human exposures, including external and internal exposure [4]. Various pathways through which people get exposed include; external irradiation, skin contamination, inhalation of dust in the form of aerosol particles, in-advertent ingestion of dirt and dust, and inhalation of radon, thoron, and their progeny through the decay products of ^238^U and ^232^Th [5].

A radioactive product is a device or manufactured item that integrates or produces radionuclides by activation, or that generates ionizing radiation that can be sold to the public without special surveillance by the competent authority Such products could either contain byproducts or source materials. However, frequent use of such products increases the risk of radiation exposure to the public. Several guidelines have been provided regarding the use of such products and the level of control exercised by the competent authorities in providing regulatory processes for exemption and how to satisfy the criteria for justification, optimization, and authorization of such products distribution to the public [6]. This is to ensure that the resulting exposure dose during its use is less than the exemption criteria, and the annual exposure dose is less than the international commission for radiation protection’s (ICRP) recommended public dose limit of 1 mSv/y. These products, which mainly contain monazite processed for use in many domestic applications and zircon (natural gemstone), are being advertised for their benefits based on radiation-induced hormesis, and the hypothesis that low-dose radiation is not only harmless but also beneficial to human health by stimulating the immune system and repair mechanism. Recent ICRP recommendations are based on the thoughtful assumption that there is no safe level of exposure, and that even the smallest amount of exposure has a chance of causing stochastic impact such as cancer. However, the basic principle of keeping all exposure levels “as low as reasonably achievable” (ALARA), in addition to staying below the dosage limits, was recommended. There is no confirmed evidence of health benefits from the use of consumer products incorporating NORMs because the available data on radiation-induced hormesis are insufficient for radiological protection studies. Therefore, it is critical to evaluate the external and internal exposure dose that the public is exposed to regarding the widespread use of such products [7]. Regarding the increased use of NORM-added consumer products, several guidelines and recommendations have been issued by notable international organizations to ensure that the radiation exposure to the public resulting from the use of such products is kept ALARA while enjoying its benefits [8]. The international atomic energy agency (IAEA) safety standard Series No. SSG 36 titled “radiation safety for consumer products” 2016, provides guidelines and suggestions for competent authorities and manufacturers of consumer goods containing small amounts of radionuclides, either intentionally introduced or emitted by activation, as well as manufacturers of radiation-generating equipment on how to satisfy the criteria for justification, optimization, and authorization of consumer product distribution to the public to ensure that the resulting dose from the use of such products does not exceed the exemption criteria specified by the competent authorities, as well the public dose limit of 1 mSv/year for low probability accident [6]. The United States Nuclear Regulatory Commission (USNRC) has identified three major areas of concern in consumer product regulation: worker radiation safety, user and public safety, and long-term environmental contamination [8]. Several kinds of consumer products containing NORMs are readily available in our daily lives. These products pose radiation risk from the inhalation of radon and thoron gases [9].

The study analyzes the external and internal exposure dose to members of the public resulting from daily use of consumer products containing naturally occurring radioactive materials and ensuring strict adherence to radiation protection principles. Because of the radioactive nature of consumer products containing NORMs, and the growing concern about the radiation risk to the public due to the widespread use of these products, the external and internal exposure of workers and members of the public should be analyzed in terms of protection quantity [10]. The European Commission’s 147 guidelines for regulatory control of consumer products containing radioactive sources classified consumer products as existing products that are currently being manufactured and readily available in the market for public use, novel products that occasionally enter the market and require authorization from the competent authority, such as glowing containing gaseous tritium light sources (GTLS) [11].

## 2. Materials and Methods

**Figure 1:**
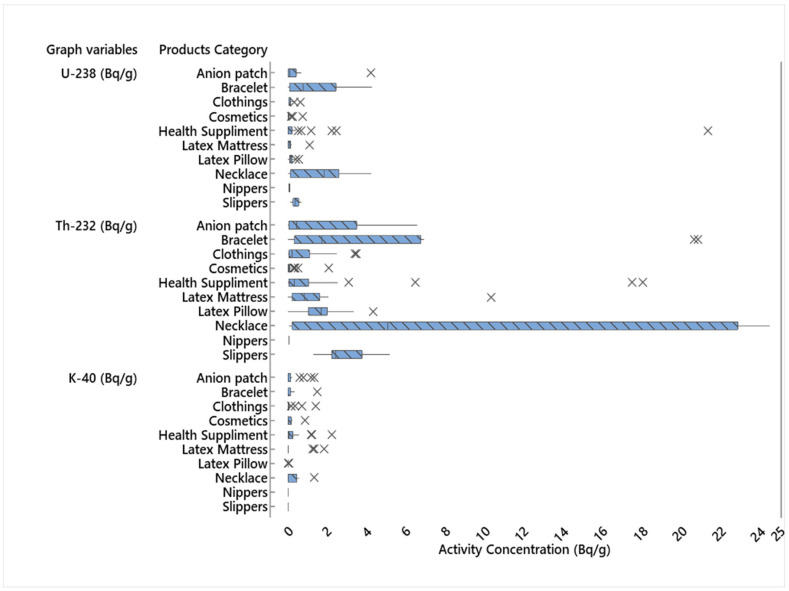
Statistical Analysis of ^238^U, ^232^Th and ^40^K using Boxplot.

For this study, data on measured activity concentrations of various consumer products containing NORMs from previous literatures and nuclear security commission (NSSC) data were obtained. The data were statistically analyzed using a boxplot, with the lower, median, and upper whiskers serving as input for dose estimation. The boxplot analysis of ^238^U, ^232^Th, and ^40^K are shown in Figure 1.

### 2.1. Hypothetical Usage Scenariosd

The hypothetical usage scenarios for various categories of consumer products incorporating NORMs were developed and categorized based on the European Union (EU) classifications of consumer products [11]. For each product category, suitable estimations were made for usage location on the body, average usage time during normal use and overuse, and exposure pathways. The average usage time for each product category during normal use was obtained from previous literature, with an additional two hours added for overuse. However, depending on the user’s behavior, usage time could differ. Table 1 presents the summary of the usage scenarios developed for this study.

### 2.2. Dose Assessment Methodology

The radiation exposure dose is categorized as external or internal depending on whether the radiation source is outside or inside the body. External dose results from direct gamma radiation or contact with any materials or products containing radiation sources, whereas internal dose results from inhalation of contaminated air or ingestion of contaminated water, food, or any products containing radioactive materials. However, applying the ICRP dose coefficient for the specific radionuclide of interest as well as other parameters is one method for estimating the external and internal exposure dose using analytical techniques.

#### 2.2.1. Effective Dose Coefficients

For radionuclides of interest, the ICRP developed dose coefficients for calculating equivalent and effective doses from inhalation and ingestion. However, when using bioassay measurement data, selecting the appropriate dose coefficient depends on the type of radionuclide, exposure pathways, particle size for inhalation, the chemical form of radionuclides, and time since intake [13]. The inhalation dose can be estimated using the activity aerodynamic diameter (AMAD), absorption type, and absorption fraction (fi). Absorption types for the human respiratory tract model are identified by particulates as fast (F), moderate (M), and slow (S) rate of solubilization, which is only applicable to inhalation dose calculation [14]. To calculate the dose from intake, multiply the total number of radionuclides inhaled or ingested by the appropriate dose coefficient, as well as other exposure parameters. Table 2 shows the age-dependent effective dose coefficient values for radionuclides inhalation and ingestion obtained from ICRP Publication 119, while external radiation exposure was extracted and calculated from ICRP 144 alongside inhalation rate from ICRP 1975, 2004, respectively.

#### 2.2.2. External Dose Assessment

The external dose comprises a dose from direct radiation or exposure from sources or material containing radioactive materials. When performing external dose assessments, several factors should be considered, including the time of exposure, distance from the source, source activity, potential shielding, and isotope. The external dose resulting from the use of NORMs added consumer products vary according to the different age groups. Although ^238^U and ^232^Th are alpha emitters, except for ^40^K, have 89% beta emitters and 11% gamma emitters, which account for the low external dose from NORM. The annual effective dose from direct radiation $\left(D_{ext.}\right)$ can be expressed as:

(1) $$D_{ext.}\left(Sv/y\right)=C_{R}\times E_{T}\times DCF_{external},\;$$

where

$$\begin{array}{c} C_{R}\;is\;the\;measured\;activity\;concentration\;of\;radionuclide\;R\;in\;Bq/g\\ E_{T}\;is\;the\;annual\;exposure\;time\;from\;external\;source\;in\;h/y\\ DCF_{external.}\;is\;the\;external\;dose\;conversion\;factor\;in\;Sv/h\;per\;Bq/g \end{array}$$

#### 2.2.3. Internal Dose Assessment

The internal dose is the amount of radiation absorbed by the body because of radioactive materials entering through various pathways. Once inside the body, the radioactive material accumulates in particular organs or tissues, delivering a radiation dose to various organs or tissues. Unlike external dose, which is caused by direct gamma radiation, the internal dose is mainly caused by inhalation and ingestion during normal use and misuse of consumer products containing NORMs and can be determined using the appropriate analytical parameters. The inhalation dose is mainly attributed to aerosols containing radioactive particles mainly radon and thoron gases, which are inhaled and deposited in the respiratory tract system. The annual effective dose from inhalation ($D_{int\left(inh\right)}$) can be calculated using the following equation as:

(2) $$D_{int\left(inh\right)}\left(mSv/y\right)=C_{R}\times I_{R}\times H_{t}\times DCF_{inh}\;,$$

where

$$\begin{array}{c} C_{R}\;is\;th\;measured\;activity\;concentration\;of\;inhaled\;radionuclide\;R\;in\;air\;\left(Bq/g\right)\\ I_{R}\;the\;inhalation\;rate\;by\;an\;individual\;decay\;products\;\left(1\;m^{3}/h\;or\;1225\;g/h\right)\\ H_{t}\;is\;the\;exposure\;time\;to\;contamninated\;air\;\left(h/y\right)\\ DCF_{inh}\;is\;the\;inhalation\;dose\;coefficient\;factor\;in\;Sv/Bq \end{array}$$

The annual effective dose from ingestion ($D_{\mathrm{int}\left(\mathrm{ing}\right)}$) can be calculated using the expression

(3) $$D_{int\left(ing\right)}\left(mSv/y\right)=C_{R}\times I_{R}\times I_{T}\times DCF_{ing},$$

where

$$\begin{array}{c} C_{R}\;is\;measured\;activity\;concentration\;of\;ingested\;radionuclide\;R\;in\;Bq/g\\ I_{R}\;\mathrm{Inadvertent}\;\mathrm{Ingestion}\;\mathrm{rate}\;g/h\\ I_{T}\;\mathrm{Time}\;\mathrm{of}\;\mathrm{Ingestion}\;\mathrm{rate}\;h/y\\ DCF_{ing}\;is\;the\;ingestion\;dose\;conversion\;factor\;in\;Sv/Bq \end{array}$$

#### 2.2.4. Total Effective Dose

The total radiation dose to the public should not exceed the ICRP’s recommended dose limit. The total annual radiation dose arising from normal and overuse of consumer products containing NORMs include all contributions arising from such product, which can be considered by calculating the total exposure dose across all pathways. Therefore, the total radiation dose (D_total_ in mSv/y) can be estimated as the sum of radiation doses from all possible exposure pathways, including direct gamma radiation from external exposure and internal exposure from inhalation and ingestion of radionuclides, and can be expressed mathematically as:

(4) $$D_{total}\left(mSv/y\right)=\;D_{ext.}+D_{int\left(inh\right)}+D_{int\left(ing\right)},$$

where:

$$\begin{array}{c} D_{total}\;is\;the\;total\;radiation\;dose\;in\;mSv/yr\\ D_{ext.}\;is\;annual\;external\;dose\;from\;direct\;gamma\;radiation\;in\;mSv/yr\\ D_{int\left(inh\right)}\;is\;annual\;internal\;dose\;due\;to\;inhallation\;pathway\;in\;mSv/yr\\ D_{int\left(ing\right)}is\;annual\;internal\;dose\;due\;to\;ingestion\;pathway\;in\;mSv/yr \end{array}$$

### 2.3. Microshield Code

MicroShield is a product of Grove software developed for photon/gamma-ray shielding and dose prediction. It is commonly used for shield design, estimating source intensity from radiation measurements, minimizing human exposure, and teaching shielding concepts. The MicroShield program uses dose conversion factors derived from ANSI/ANS-6.1.1-1977 standards. The standard has been used for radiation shielding measurements to measure whole-body exposure rates for radiation workers and the public [18]. MicroShield calculates gamma-ray shielding using the point kernel approach. That is, the source region is first divided into several small volumes, then the dose contribution of each volume portion is incorporated into space and energy in the calculation. Other pertinent properties, such as the mass attenuation coefficient, atomic radius of nuclides, and shield materials used in the calculation were used as default values in the code.

### 2.4. Integrated Module for Bioassay Analysis (IMBA) Code

Integrated Module for Bioassay Analysis (IMBA) software was developed by Public Health England to evaluate the internal exposure doses that can occur from radionuclides intakes or bioassay measurement data in various scenarios. For various internal dose calculations from inhalation, ingestion, injection, wound, and vapor routes, the code uses the ICRP dose coefficient and other parameters of ICRP publications 26, 30, 56, 66, 67, 68, and 69, as well as the National Council on Radiation Protection (NCRP) 156 and Code of Federal Regulation (10CFR 835) [19]. In bioassay and dose calculations, it uses biokinetic and dosimetric models, respectively.

### 2.5. Visual Monte Carlo (VMC) Code

Visual Monte Carlo (VMC) software was developed by John Hunt at the Instituto de Radio-protecao e Dosimetria (France) in two versions: VMC in vivo for modeling of Whole Body Counter laboratories, and VMC dose calculation for dose calculations due to radionuclide or X-ray exposure [20]. With VMC dose computation, Monte Carlo estimates for exposure to photon fields generated by radionuclides or X-ray equipment are possible. The program Visual Basic Programming with a graphics user interphase (GUI) is particularly useful for calculating doses in the event of an accident or an emergency in which high-activity sources are placed close to the body, by calculating the tissue equivalent dose to each radiosensitive organ as defined in the ICRP 103 recommendation, and it also allows isodose curves to be established in the region close to the source using ICRP voxel phantoms of humans. The comparison of the various computer codes used for estimating both external and internal dose is presented in Table 3.

## 3. Results

### 3.1. Age-Dependent Dose Calculation

**Figure 2:**
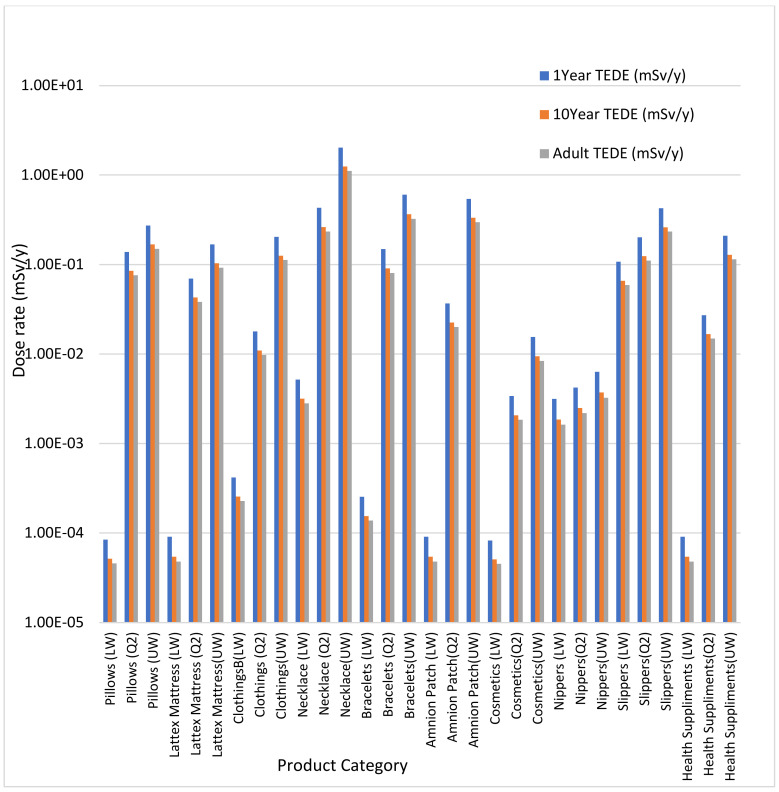
Age-dependent total effective dose equivalent.

For all age groups and input ranges considered, the result of age-dependent for external and internal dose calculation using the analytical solution in Table S1 shows low dose reported for external dose assessment from measured activity concentration of consumer products incorporating NORM. Internal dose results are higher than external dose and continue to increase as the range of input parameters increases from lower whiskers to median and upper whiskers. The dose reported for internal exposure are high due to gaseous decay of ^238^U and ^232^Th (radon and thoron) which are harmful when inhaled. Low dose reported for external exposure because ^40^K decays by emitting 89% beta emitter and 11% gamma emitter, thus, contribution to external dose are due to 11% gamma decay of ^40^K. Higher internal dose values of 2.03 mSv/y, 1.24 mSv/y, and 1.11 mSv/y reported for 1 year old, 10 year old, and adult age groups, due to high activity concentrations of ^238^U (4.21 Bq/g), ^232^Th (24.1 Bq/g), and ^40^K (0.55 Bq/g) in the upper whiskers of the necklace. The dose reported is higher for one-year-olds than ten-year-olds and adults because growing tissues are more sensitive to radiation than a matured tissue. The high dose is also attributed due to position of necklace close to the nose which makes it possible for inhalation of gaseous decay products of ^238^U and ^232^Th, respectively. The total effective dose equivalent for various age groups considered in this study are presented in Figure 2. The TEDE for various age groups reported at the upper whisker of necklace with an activity concentration of 4.21 Bq/g (^238^U), 24.1 Bq/g (^232^Th), and 0.55 Bq/g (^40^K) are 2.03 mSv/y, 1.24 mSv/y, and 1.11 mSv/y, for one-year-olds, ten-year-old, and adult age groups, respectively, which are all above the ICRP recommended public dose limit of 1 mSv/y.

### 3.2. Internal Dose Calculation Using IMBA Code

**Figure 3:**
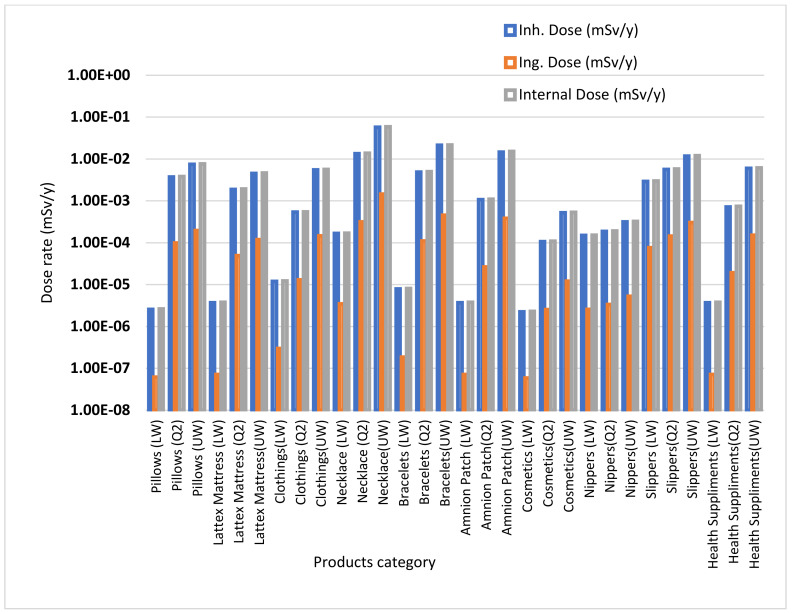
Internal dose from inhalation and ingestion using IMBA code.

Internal doses from inhalation and ingestion were calculated using IMBA code and the result is presented in Figure 3. The internal dose from inhalation is higher than that from ingestion for all products due to inhalation of radon and thoron gas emitted by the decay product of ^238^U and ^232^Th. Contribution of ingestion dose is negligible since it is hard to ingest such products directly but inhalation of gaseous decay products is possible.

### 3.3. External Dose Using Microshield Code

**Figure 4:**
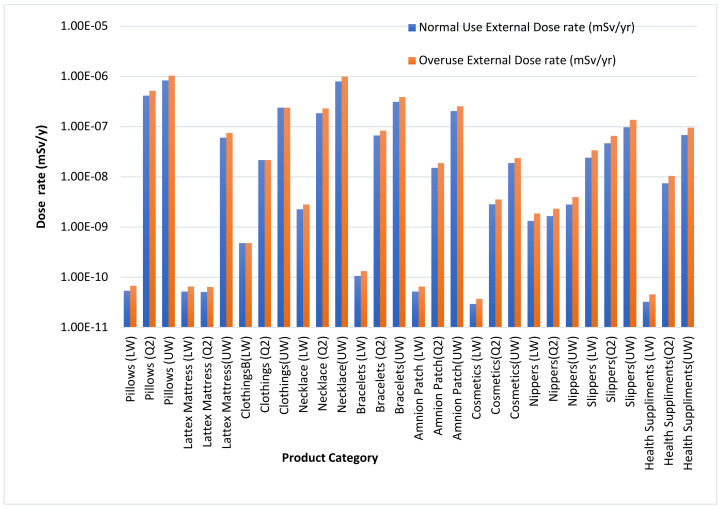
External dose using Microshield code.

External doses were evaluated using Microshield computer code for normal use and overuse, and the result is presented in Figure 4. The external doses for normal use and overuse are all below the ICRP public dose limit of 1 mSv/y. Low external dose is due to 11% contribution of gamma decay during ^40^K decay. Although doses reported for both usages are low, doses encountered during overuse scenario are higher than the normal use scenario.

### 3.4. Visual Monte Carlo (VMC) Code

**Figure 5:**
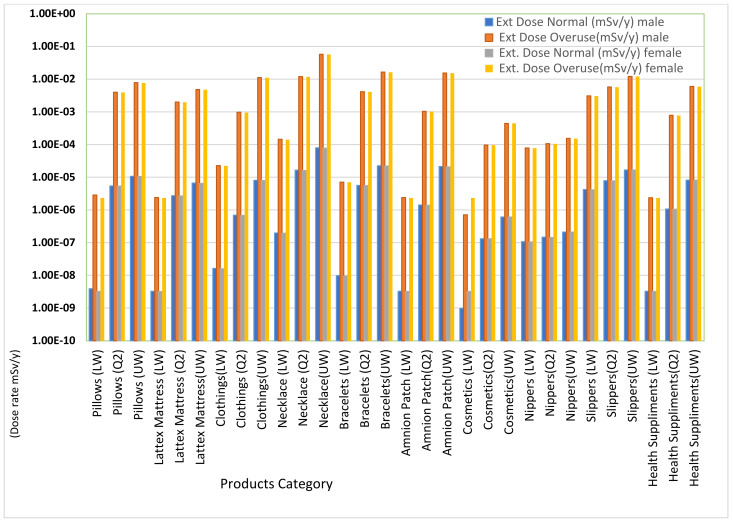
External dose during normal use and overuse for both phantoms using VMC.

**Figure 6:**
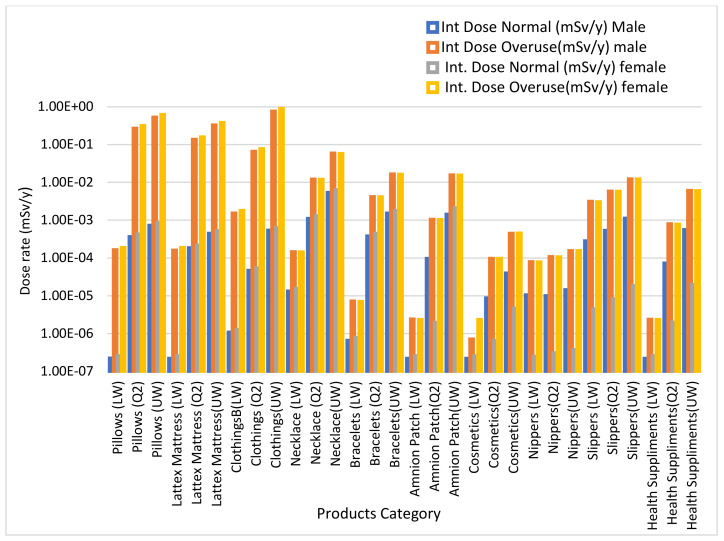
Internal dose during normal use and overuse for both phantoms VMC.

External dose for male and female phantom during normal use and overuse is presented in Figure 5. Although the eternal doses reported for male and female phantom during normal use and overuse are all low, doses during overuse are all higher than the dose during normal use for all ranges of data from both phantoms. Low external doses are attributed to 11% gamma decay of ^40^K. Calculations were performed using both male and female phantoms due to differences in organs and their sensitivity to radiation. Figure 6 shows the internal dose from male and female phantom during normal use and overuse. Even though the internal doses reported for both phantoms during normal use and overuse are all low, this dose are higher than the external dose reported in Figure 5 for both phantoms. The internal dose is attributed to the inhalation of gaseous decay products of ^238^U and ^232^Th, which are harmful when inhaled.

### 3.5. Code Comparison for External and Internal Dose Calculations

**Figure 7:**
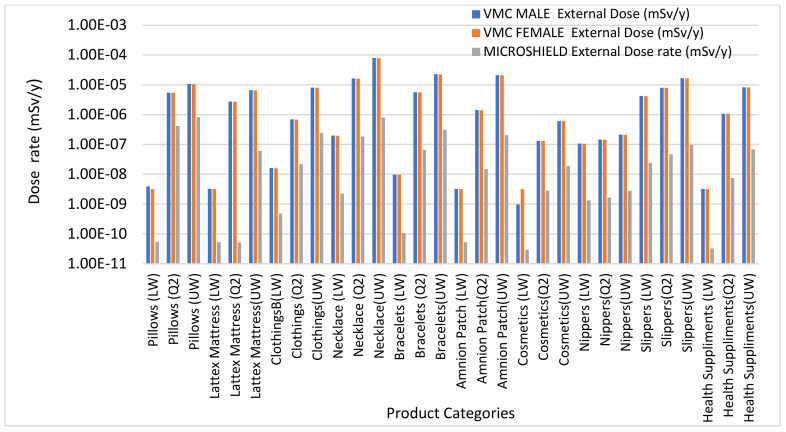
Comparison of external dose using VMC Phantoms and Microshield code.

**Figure 8:**
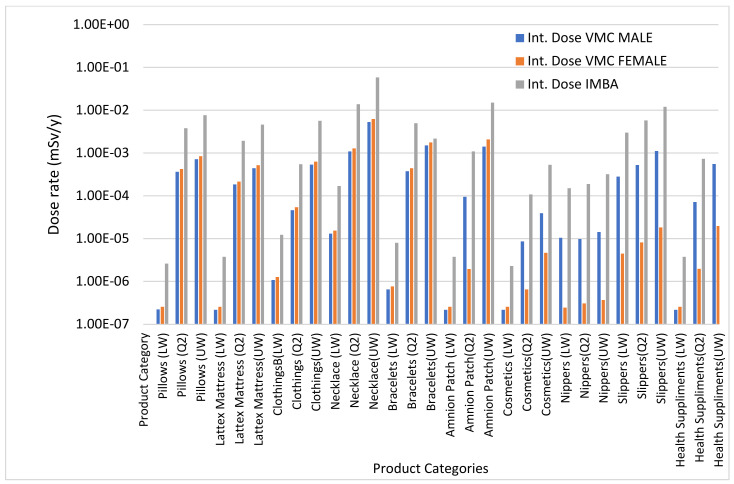
Comparison of internal dose using VMC phantoms and IMBA code.

Figure 7 and Figure 8 show the results of code comparison for external and internal dose calculation using Microshield, VMC male and female Voxel Phantom, and IMBA. The comparison for external dose using VMC phantoms and Microshield is shown in Figure 7. The external dose calculated using Microshield is lower than VMC male and female voxel phantoms. The difference in results could be due to the uncertainty range of associated with each computer code. Additionally, the difference could also be due to sensitivity in organs for both phantoms, little variability was observed using VMC male and female phantom. In all cases, the dose obtained using Microshield is lower than the dose obtained using VMC. Figure 8 shows comparison of internal dose results obtained using VMC phantoms and IMBA code. For all categories and ranges of input data used, the internal dose assessment using VMC phantoms is lower than IMBA Code. Although IMBA is a famous internal dosimetry tool, the differences in the results could be due to uniqueness and uncertainty range associated with each computer code.

## 4. Discussion

For various ranges of input data used, the result of age-dependent dose calculation using the analytical solution shows a low dose reported for external dose assessment from measured activity concentration of consumer products containing NORMs. Because of alpha (α) decay of ^238^U and ^232^Th which is dangerous when inhaled, and ^40^K decay by emitting 89% beta (β) and 11% gamma, thus, majority of the external dose results from contribution of gamma decay of ^40^K. Higher internal doses of 2.03 mSv/y, 1.24 mSv/y, and 1.11 mSv/y were obtained in 1 year old, 10 year old and adult groups, due to high activity concentrations of ^238^U (4.21 Bq/g), ^232^Th (24.1 Bq/g), and ^40^K (0.55 Bq/g), reported at the upper whiskers of the necklace. These doses are all above the ICRP recommended public dose limit of 1 mSv/y. The dose is higher in one-year-old children than in ten-year-olds and adults due to sensitivity of a growing tissue to radiation than a mature tissue. Though it is not much likely that a one-year old child wears a necklace all day long for one year, however, analytical dose assessment was performed for various age groups based on the assumption of annual usage time for all product categories to see the effects of radiation sensitivity on various tissues. High internal doses are also attributed due to the proximity of necklace to the nose which makes it possible and easy for inhalation of gaseous decay products of ^238^U and ^232^Th as the most dominant pathway. In addition, the TEDE reported at the upper whicker of the necklace for various age groups are all above the ICRP recommended public dose limit of 1 mSv/y. Results of internal doses from inhalation are higher than that of doses from ingestion in all categories due to inhalation of gaseous decay product of ^238^U and ^232^Th, with low tendency of ingestion dose during usage of consumer products incorporating NORMs. Although low eternal doses reported for both phantoms during normal use and overuse are due to contribution from 11% gamma decay of ^40^K, the dose during overuse are all higher than the dose during normal use in all cases. Similarly, internal doses reported using VMC code for both phantoms during normal use and overuse are all below the recommended dose limit of 1 mSv/y. The major exposure pathway for internal dose is inhalation pathway. Because of the uncertainty range of the code and differences in organs for both phantoms, external dose obtained using Microshield are lower than the dose obtained using VMC phantoms. Comparison of internal dose assessment shows that the doses obtained using VMC phantoms are lower than the doses obtained using the IMBA code in all cases. This is because of the unique uncertainty range associated with each computer code. Regardless, IMBA has been validated as a famous internal dosimetry code which makes is more preferred than VMC [21]. Comparison of external dose assessment shows that the dose calculated using Microshield is lower than VMC male and female voxel phantoms. Thus, Microshield is more preferred to VMC because of its wide range of application in external dose assessment. In the previous studies, various voxel phantoms were used to evaluate external doses. However, in this study, both external and internal doses, as well as TEDE were evaluated for various age groups using normalized activity concentration, by applying both analytical calculation and computer codes, comparisons of results for external and internal dose obtained were made using various computer codes.

## 5. Conclusions

The ICRP dose limits are designed to serve as a boundary condition to prevent deterministic consequences while limiting the possibility of stochastic effects. Doses more than 1 mSv/y will necessitate the implementation of public safety measures. In this study, radiological dose assessment from consumer products containing NORMs was performed using various usage scenarios and ranges of input data for each product category. The data in this study were analyzed using Boxplot with lower whiskers, median and upper whiskers used as input for analysis. A normalized activity concentration values was used to perform external and internal dose assessment using analytical calculation by applying ICRP dose coefficients and other input parameters, VMC, Microshield, and IMBA computer codes were also applied, and code comparison was made. The total effective dose equivalent was evaluated using a normalized activity concentration of NORMs and age-dependent dose coefficients for three age groups using analytical calculation. The results show that the highest TEDE received from necklace products with normalized activity concentrations of 4.21 Bq/g (^238^U), 24.4 Bq/g (^232^Th), and 0.55 Bq/g (^40^K) for various age groups considered in this study are 2.03 mSv/y for one-year-olds, 1.24 mSv/y for ten-year-olds and 1.11 mSv/y for adults, respectively, which are all above the recommended public dose limit of 1 mSv/y as recommended by ICRP. Dose evaluation results obtained for external and internal exposure using VMC phantoms, Microshield, and IMBA code are all below the recommended public dose limit of 1 mSv/y. Microshield and IMBA codes are found to be the most preferred computer codes for external and internal dosimetry than VMC considering their wide range of applications.

## Figures and Tables

**Table 1:** Summary of hypothetical usage scenarios and exposure pathways.

Product Categories	EU Classifications	Usage Location	Average Usage Time (per Day)		Assumed Condition for Overuse	Exposure Pathways
			Normal Use [12]	Overuse Day		
Pillows	Existing Products	Head, neck	7 h 50 min	9 h 50 min	Sickness/oversleeping	Inhalation
Latex Mattress	Existing Products	Whole body	7 h 50 min	9 h 50 min	Sickness/oversleeping	Inhalation
Clothing	Existing Products	Depending on usage	24 h	1 day 2 h	overuse	Inhalation
Necklace	Existing Products	Neck	8 h 7 min	10 h 7 min	overuse	Inhalation
Bracelets	Existing Products	Hand	8 h 7 min	10 h 7 min	overuse	Inhalation
Amnion Patch	Existing Products	Body for wound covering	8 h 7 min	10 h 7 min	overuse	Inhalation and ingestion
Cosmetics	Existing Products	Face and body	8 h 7min	10 h 7 min	Accidental Ingestion	Ingestion
Dippers	Existing Products	Depending on usage	20 min	2 h 20 min	overuse	Inhalation
Slippers	Existing Products	Foot wares	5 h 1 min	7 h 1 min	overuse	Inhalation
Health Supplements	Existing Products	Waist, abdomen, etc.	5 h 1 min	7 h 1 min	overuse	External and inhalation

**Table 2:** Age-dependent dose coefficients and inhalation rate.

Nuclide	T_1/2_	Effective Dose Coefficient for Inhalation (Sv/Bq) [15].				
		Type	fi	1 Year Old	10-Year-Old	Adult
^238^U	4.468 × 10^9^	F	0.02	1.3 × 10^−6^	7.3 × 10^−4^	5.0 × 10^−7^
		M	0.02	9.4 × 10^−6^	4.0 × 10^−4^	2.9 × 10^−6^
		S	0.002	2.5 × 10^−6^	1.0 × 10^−4^	8.0 × 10^−6^
^232^Th	1.405 × 10^1^	F	0.0005	2.2 × 10^−6^	1.3 × 10^−4^	1.1 × 10^−4^
		M	0.0005	8.1 × 10^−5^	5.0 × 10^−5^	4.5 × 10^−5^
		S	0.0005	5.0 × 10^−5^	2.6 × 10^−5^	2.5 × 10^−5^
^40^K	1.28 × 10^9^	F	1.0	1.7 × 10^−8^	4.5 × 10^−9^	2.1 × 10^−9^
**Effective Dose Coefficient for Ingestion (Sv/Bq) [15].**						
^238^U	4.468 × 10^9^	-	0.02	1.2 × 10^−7^	6.8 × 10^−8^	4.5 × 10^−8^
^232^Th	1.405 × 10^1^	-	0.0005	4.5 × 10^−7^	2.9 × 10^−7^	2.3 × 10^−7^
^40^K	1.28 × 10^9^	-	1.0	4.2 × 10^−8^	1.3 × 10^−8^	6.2 × 10^−9^
**Effective Dose Coefficient for External Exposure extracted and calculated from ICRP 144) (Sv/h per Bq/g) [16].**						
^238^U	4.468 × 10^9^	-	-	1.48 × 10^−16^	1.15 × 10^−16^	9.44 × 10^−17^
^232^Th	1.40 × 10^1^	-	-	5.06 × 10^−16^	4.02 × 10^−16^	3.37 × 10^−16^
^40^K	1.28 × 10^9^	-	-	4.39 × 10^−13^	3.79 × 10^−13^	3.46 × 10^−13^
**Age-dependent Inhalation rate extracted from ICRP recommendations 1975, 2004 [17].**						
Unit	Inhalation rate of 1 m^3^/h or 24 m^3^/d equivalents to 1225 g/h					
m^3^/d	-	-	-	5.1	15.2	22.2
m^3^/hr	-	-	-	0.2125	0.63	0.925
g/hr	-	-	-	260	775	1331.13

**Table 3:** Computer code comparison, models and assumptions.

Code	Models	Assumptions	Purpose	Scope
IMBA	Dosimetric	ICRP Publications 60 and 68, 26 and 30 or 10CFR 835	Dosimetric calculation	Internal dose calculation
	Biokinetic		Bioassay Sample calculation	
Microshield	ANSI/ANS-6.1.1-1977 standards	Point kernel	Calculating gamma-ray shielding and dose prediction.	External dose calculation
VMC	In vivo bioassay	Monte Carlo method for radiation transport and human body voxel simulator with ICRP 110 adult male and female computational voxel phantoms	Calculating the calibration factor of energy	Estimating both external and internal dose using ICRP Voxel male and female phantom
	Dose calculation		Calculating tissue and effective doses for geometry and radionuclides	

## Data Availability

The data that support the findings of this study are available on request from the corresponding author.

## Supplementary Materials

The following are available online at https://www.mdpi.com/article/10.3390/ijerph18147337/s1, Table S1: age-dependent external and internal dose using analytical calculation.
