# Supplementary material for: Radiological Dose Assessment to Members of the Public Using Consumer Products Containing Naturally Occurring Radioactive Materials in Korea
Source: Int J Environ Res Public Health. 2021 Jul 9;18(14):7337. doi: 10.3390/ijerph18147337 (PMC8303814; doi:10.3390/ijerph18147337)
Supplement: Supplementary file 1 [file ijerph-18-07337-s001.zip › ijerph-1273361-supplementary.pdf]

Supplementary Materials:

Table 1. Age-dependent external and internal dose using analytical calculation.

Product Category	External Dose (mSv/y)			Internal Dose (mSv/y)		
	1Year	10Year	Adult	1Year	10Year	Adult
Pillows (LW)	1.61×10^{-12}	1.27×10^{-12}	1.07×10^{-12}	8.43×10^{-5}	5.15×10^{-5}	4.59×10^{-5}
Pillows (Q2)	2.54×10^{-9}	2.02×10^{-9}	1.69×10^{-9}	1.38×10^{-1}	8.51×10^{-2}	7.60×10^{-2}
Pillows (UW)	5.05×10^{-9}	4.01×10^{-9}	3.36×10^{-9}	2.73×10^{-9}	1.68×10^{-1}	1.50×10^{-1}
Matress (LW)	1.91×10^{-12}	1.51×10^{-12}	1.26×10^{-12}	9.10×10^{-5}	5.44×10^{-5}	4.79×10^{-9}
Matress (Q2)	1.28×10^{-9}	1.02×10^{-9}	8.54×10^{-10}	6.96×10^{-2}	4.28×10^{-2}	3.83×10^{-2}
Matress (UW)	1.85×10^{-8}	2.46×10^{-12}	1.42×10^{-8}	1.68×10^{-1}	1.03×10^{-1}	9.23×10^{-2}
Clothings (LW)	2.35×10^{-11}	1.86×10^{-11}	1.56×10^{-11}	4.17×10^{-4}	2.56×10^{-4}	2.28×10^{-4}
Clothings (Q2)	1.02×10^{-9}	8.09×10^{-10}	6.77×10^{-10}	1.79×10^{-2}	1.09×10^{-2}	9.75×10^{-3}
Clothings (UW)	4.61×10^{-7}	8.97×10^{-9}	3.62×10^{-7}	2.04×10^{-1}	1.26×10^{-1}	1.12×10^{-1}
Necklace (LW)	1.01×10^{-11}	7.97×10^{-11}	6.66×10^{-11}	5.20×10^{-3}	3.17×10^{-3}	2.81×10^{-3}
Necklace (Q2)	2.62×10^{-8}	6.57×10^{-9}	1.96×10^{-8}	4.30×10^{-1}	2.62×10^{-1}	2.33×10^{-1}
Necklace (UW)	7.43×10^{-7}	3.01×10^{-8}	5.81×10^{-7}	*2.03* $\times 10^{0}$	*1.24* $\times 10^{0}$	*1.11* $\times 10^{0}$
Bracelets (LW)	4.86×10^{-12}	3.86×10^{-12}	3.23×10^{-12}	2.54×10^{-4}	1.55×10^{-4}	1.38×10^{-4}
Bracelets (Q2)	2.90×10^{-9}	2.30×10^{-9}	1.92×10^{-9}	1.49×10^{-1}	9.06×10^{-2}	8.05×10^{-2}
Bracelets (UW)	4.38×10^{-7}	9.52×10^{-9}	3.43×10^{-7}	6.02×10^{-1}	3.64×10^{-1}	3.23×10^{-1}
Amnion Patch (LW)	1.91×10^{-12}	1.51×10^{-12}	1.26×10^{-12}	9.10×10^{-5}	5.44×10^{-5}	4.79×10^{-5}
Amnion Patch (Q2)	1.93×10^{-8}	5.49×10^{-10}	1.51×10^{-8}	3.68×10^{-2}	2.25×10^{-2}	2.01×10^{-2}
Amnion Patch (UW)	2.51×10^{-7}	7.92×10^{-9}	1.97×10^{-7}	5.40×10^{-1}	3.32×10^{-1}	2.97×10^{-1}
Cosmetics (LW)	1.52×10^{-12}	1.21×10^{-12}	1.01×10^{-12}	8.24×10^{-5}	5.07×10^{-5}	4.53×10^{-5}
Amnion Patch (Q2)	3.34×10^{-8}	5.17×10^{-11}	2.63×10^{-9}	3.39×10^{-3}	2.07×10^{-3}	1.84×10^{-3}
Amnion Patch (UW)	2.62×10^{-7}	2.41×10^{-9}	2.06×10^{-7}	1.55×10^{-02}	9.42×10^{-3}	8.36×10^{-3}
Nippers (LW)	4.37×10^{-11}	3.45×10^{-11}	4.09×10^{-11}	3.15×10^{-3}	1.86×10^{-3}	1.63×10^{-3}
Nippers (Q2)	5.70×10^{-11}	4.50×10^{-11}	5.41×10^{-11}	4.21×10^{-03}	2.49×10^{-3}	2.19×10^{-3}
Nippers (UW)	8.97×10^{-11}	7.08×10^{-11}	8.29×10^{-11}	6.34×10^{-3}	3.72×10^{-3}	3.24×10^{-3}
Slippers (LW)	1.24×10^{-9}	9.83×10^{-10}	8.24×10^{-10}	1.07×10^{-3}	6.59×10^{-2}	5.89×10^{-2}
Slippers (Q2)	2.35×10^{-9}	1.86×10^{-9}	1.56×10^{-9}	2.02×10^{-1}	1.24×10^{-3}	1.11×10^{-1}
Slippers (UW)	4.92×10^{-9}	3.91×10^{-9}	3.27×10^{-9}	4.25×10^{-1}	2.61×10^{-91}	2.33×10^{-1}
Health Sup. (LW)	1.19×10^{-12}	9.44×10^{-13}	7.87×10^{-13}	9.10×10^{-5}	5.44×10^{-5}	4.79×10^{-5}
Health Sup. (Q2)	3.72×10^{-8}	2.47×10^{-10}	2.93×10^{-8}	2.71×10^{-5}	1.67×10^{-2}	1.49×10^{-2}
Health Sup (UW)	4.48×10^{-7}	1.94×10^{-9}	3.53×10^{-7}	2.09×10^{-1}	1.28×10^{-1}	1.15×10^{-1}
